# Complete Plastid Genome Sequences of Four Salsoleae s.l. Species: Comparative and Phylogenetic Analyses

**DOI:** 10.3390/biom14080890

**Published:** 2024-07-24

**Authors:** Shyryn Almerekova, Moldir Yermagambetova, Bektemir Osmonali, Polina Vesselova, Yerlan Turuspekov, Saule Abugalieva

**Affiliations:** 1 Molecular Genetics Laboratory, Institute of Plant Biology and Biotechnology, Almaty 050040, Kazakhstan; almerekovakz@gmail.com (S.A.); ermaganbetova.moldir@bk.ru (M.Y.); yerlant@yahoo.com (Y.T.); 2 Faculty of Biology and Biotechnology, Al-Farabi Kazakh National University, Almaty 050040, Kazakhstan; 3 Laboratory of the Flora of Higher Plants, Institute of Botany and Phytointroduction, Almaty 050040, Kazakhstan; be96ka_kz@mail.ru (B.O.); pol_ves@mail.ru (P.V.)

**Keywords:** Illumina sequencing, Salsoleae s.l., Kazakhstan, plastid genome comparison, polymorphic regions, phylogenetic relationships

## Abstract

The taxonomic classification of the genera *Salsola* L., *Pyankovia* Akhani and Roalson, and *Xylosalsola* Tzvelev within Chenopodiaceae Vent. (Amaranthaceae s.l.) remains controversial, with the precise number of species within these genera still unresolved. This study presents a comparative analysis of the complete plastid genomes of *S. foliosa*, *S. tragus*, *P. affinis*, and *X. richteri* species collected in Kazakhstan. The assembled plastid genomes varied in length, ranging from 151,177 bp to 152,969 bp for *X. richteri* and *S. tragus*. These genomes contained 133 genes, of which 114 were unique, including 80 protein-coding, 30 tRNA, and 4 rRNA genes. Thirteen regions, including *ndhC-ndhD*, *rps16-psbK*, *petD*, *rpoC2*, *ndhA*, *petB*, *clpP*, *atpF*, *ycf3*, *accD*, *ndhF-ndhG*, *matK*, and *rpl20-rpl22*, exhibited relatively high levels of nucleotide variation. A total of 987 SSRs were detected across the four analyzed plastid genomes, primarily located in the intergenic spacer regions. Additionally, 254 repeats were identified, including 92 tandem repeats, 88 forward repeats, 100 palindromic repeats, and only one reverse repeat. A phylogenetic analysis revealed clear clustering into four clusters corresponding to the Salsoleae and Caroxyloneae tribe clades. These nucleotide sequences obtained in this study represent a valuable resource for future phylogenetic analyses within the Salsoleae s.l. tribe.

## 1. Introduction

The Chenopodiaceae Vent. (Amaranthaceae s.l.) family, also known as the goosefoot family, encompasses diverse plant species distributed worldwide, particularly in arid and semi-arid regions. Chenopodiaceae is one of the largest families, comprising approximately 110 genera and over 1700 species [1]. The representatives of this family play significant ecological roles as pioneer colonizers of disturbed habitats and providers of forage for wildlife and livestock [2]. One of the largest and most economically important genera within the family is *Salsola* L. [3]. The precise number of *Salsola* species worldwide is uncertain, with estimates ranging from 130 to 150, depending on the different taxonomic revisions [3,4,5,6,7]. These species are widely distributed across desert and semi-desert regions spanning Central Asia, the Middle East, Africa, and Europe [8,9]. The taxonomy of the tribe Salsoleae s.l., which includes the genus Salsola, remains controversial, and the exact number of genera within the tribe is still under debate [10]. Akhani et al. [10] revised the classification of Salsoleae s.l. based on nuclear ribosomal internal transcribed spacer (*ITS*) and chloroplast *psbB-psbH* markers. Their findings revealed that three genera are newly described (*Pyankovia, Kaviria*, and *Turania*), while four previously recognized genera (*Caroxylon, Climacoptera*, *Kali*, and *Xylosalsola*) have been resurrected [10]. Another extensive phylogenetic study focused on Salsoleae s.l. taxa from northwestern China, and employed nucleotide sequences from *ITS, psbB–psbH*, and *rbcL* [11]. The authors [11] reported that the tribe Salsoleae s.l. is monophyletic and consists of three distinct monophyletic subunits: Caroxyloneae, the *Kali* clade, and s.str. The genus *Xylosalsola* Tzvelev was formerly considered a section within *Salsola* before being recognized as an independent genus [10]. According to POWO (Plants of the World Online) [12], *Xylosalsola* is represented by four accepted species, with its native range extending from South European Russia to Mongolia and Pakistan. The newly recognized genus *Pyankovia* Akhani and Roalson is naturally found across territories from Krym to Mongolia and Afghanistan [12], comprising three distinct species. The species within this genus were previously classified as belonging to *Climacoptera* Botsch. [10]. The species from these genera play a dominant role in forming various plant communities in dry climates [13,14]. They demonstrate adaptability to different environmental conditions, such as saline soils and harsh climates, providing forage for livestock in such regions [15,16]. Furthermore, the plants of *Salsola tragus* L. and *Xylosalsola rechteri* (Moq.) Akhani and Roalson are widely used in folk medicine [17,18]. Despite their ecological, economic, and medicinal significance, ongoing research and debate persist regarding the taxonomic classification and phylogenetic relationships within these genera belonging to the tribe Salsoleae s.l.

In addition to the numerous studies on the taxonomy of Salsoleae s.l. [19,20,21,22], population genetics analyses have been conducted by employing diverse DNA markers. The genetic diversity of *Salsola* species populations has been investigated using inter-simple sequence repeat, or ISSR [23,24]; amplified length polymorphism, or AFLP [25]; and simple sequence repeat, or SSR [26,27], markers. In prior investigations [26,27], SSR markers derived from *Beta* were utilized for cross-genera amplification in *Salsola* species due to the absence of species-specific SSRs designed for *Salsola*. Furthermore, SSRs specific to *Xylosalsola* and *Pyankovia* species are currently unavailable.

Chloroplasts play a crucial role in photosynthesis in plants and possess their own genome [28]. Structurally, the genome of chloroplasts typically maintains a highly conserved quadripartite organization in angiosperms, characterized by a pair of inverted repeats (IRs) flanking a large single-copy (LSC) region and a small single-copy (SSC) region [29]. Recent advances in sequencing technologies have facilitated the rapid and cost-effective acquisition of nucleotide sequences of plastomes [30]. Plastid genomes’ polymorphic regions within nucleotide sequences can be utilized to generate DNA-barcoding markers across different taxonomic levels [31]. The SSRs extracted from plastid genomes may serve as valuable molecular markers for population and conservation genetics [32,33]. The highly conserved nature of chloroplast SSRs allows for cross-species amplification in the same family [34,35,36]. Because of its small genome size and conserved structure, the plastid genome is extensively utilized in phylogenetic analyses across various plant species [28,37].

Research on the plastid genomes of *Salsola*, *Xylosalsola*, and *Pyankovia* is limited, with only the plastid genome of *Salsola abrotanoides* having been characterized [38]. In this study, we report a comparative analysis of the complete plastid genome of four species, *Salsola foliosa* (L.) Schrad. ex Schult., *Salsola tragus* L., *Pyankovia affinis* (C.A.Mey. ex Schrenk) Mosyakin and Roalson, and *Xylosalsola richteri* (Moq.) Akhani and Roalson, collected in Kazakhstan. The obtained results may serve as a valuable resource for future studies on population genetics and phylogenetic analyses of species within the tribe Salsoleae s.l.

## 2. Materials and Methods

### 2.1. Plant Materials and DNA Extraction

**Figure 1:**
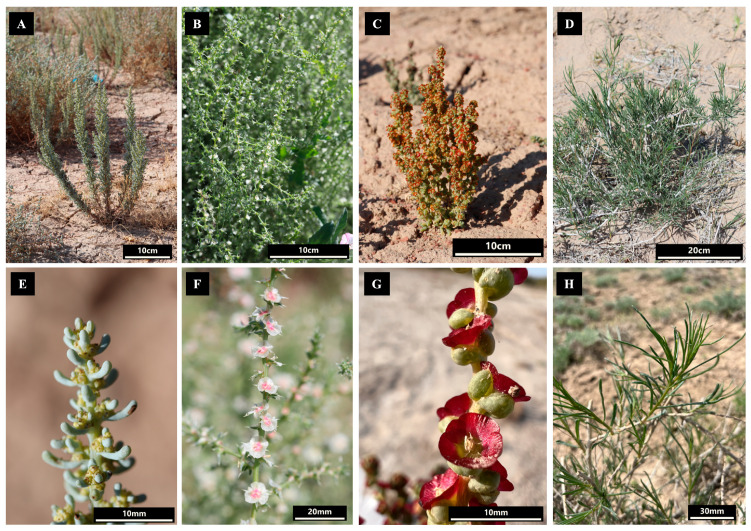
Pictures of *Salsola foliosa*, *Salsola tragus*, *Pyankovia affinis*, and *Xylosalsola richteri* in natural conditions ((**A**–**D**), respectively) and close-up representations of *S. foliosa*, *S. tragus*, *P. affinis*, and *X. richter* ((**E**–**H**), respectively).

Fresh leaves were harvested from mature plants of the *Salsola foliosa*, *Salsola tragus*, *Pyankovia affinis*, and *Xylosalsola richteri* species (Figure 1). Each species’ fresh leaves were promptly dehydrated using silica gel to facilitate subsequent DNA extraction. The collection location and GPS coordinates are detailed in Table 1. The specimens of *S. foliosa*, *S. tragus, P. affinis*, and *X. richteri* were deposited in the herbarium (AA) of the Institute of Botany and Phytointroduction. Genomic DNA was isolated from the dried leaves by utilizing the cetyltrimethylammonium bromide (CTAB) protocol [39]. The genomic DNA quality was assessed using 1% agarose gel electrophoresis and quantified using the NanoDrop™ One spectrophotometer (Thermofischer, Waltham, MA, USA).

### 2.2. Library Construction and De Novo Sequencing

Genomic DNA that met quality control standards was utilized for library preparation by employing the TruSeq Nano DNA Kit (Illumina Inc., San Diego, CA, USA). Subsequently, genome sequencing was conducted on the Illumina NovaSeq 6000 platform by employing a 151 bp paired-end read (Macrogen Inc., Seoul, Republic of Korea).

### 2.3. Genome Assembly and Annotation

The initial quality control checks on the raw sequence data were performed using FastQC (http://www.bioinformatics.babraham.ac.uk/projects/fastqc, accessed on 29 January 2024). The Trimmomatic 0.36 software [40] was employed to eliminate adapter sequences from the raw reads, followed by the assembly of the clean reads using NOVOPlasty 4.3.3 [41]. The assembled plastid genomes were annotated using GeSeq (https://chlorobox.mpimp-golm.mpg.de/geseq.html, accessed on 22 February 2024) [42] and based on comparisons with the plastid genomes of *Salsola affinis* (ON080842), *Salsola abrotanoides* (MW123092) [38], and *Caroxylon passerinum* (MW192441) [43]. A graphical map of the annotated circular plastid genome was generated using the OrganellarGenomeDRAW tool 1.3.1 (OGDRAW) [44]. The annotated plastome sequences of the analyzed species were submitted to GenBank.

### 2.4. Simple Sequence Repeats (SSRs) and Long Repetitive Sequence Analysis

SSRs were detected using the web-based tool MISA (https://webblast.ipk-gatersleben.de/misa/, accessed on 8 May 2024) [45], with the minimum repeat number set to 8, 4, 4, 3, 3, and 3 for mononucleotides, dinucleotides, trinucleotides, tetranucleotides, pentanucleotides, and hexanucleotides, respectively. Forward (F), reverse (R), and palindromic (P) repeats were identified using REPuter (https://bibiserv.cebitec.uni-bielefeld.de/reputer/, accessed on 8 May 2024) [46] with minimal repeats exceeding 30 bp and Hamming distances of less than 3 bp. Additionally, tandem repeats (T) were identified using the Tandem repeats finder (https://tandem.bu.edu/trf/trf.html, accessed on 10 May 2024) [47] tool with the default settings.

### 2.5. Nucleotide Diversity and Ka/Ks Ratio Analysis

Sequences of protein-coding genes were aligned using Geneious Prime^®^ 2024.0.2 (https://www.geneious.com, accessed on 10 May 2024) to assess the nucleotide diversity (Pi) of the protein-coding genes across the 13 analyzed samples. Subsequently, a sliding window analysis was conducted to determine the nucleotide variability (Pi) values using DnaSP 6 [48], employing a window length of 600 bp and a step size of 200 bp. We separately isolated and aligned the 80 protein-coding genes of 13 samples to evaluate the synonymous (Ks) and nonsynonymous (Ka) substitution rates. The Ka/Ks ratios for each gene were then analyzed using DnaSP 6 [48].

### 2.6. Phylogenetic Analyses

A phylogenetic analysis was carried out using the nucleotide sequences of a complete plastid genome sourced from 13 samples of 12 species aligned using Geneious Prime^®^ 2024.0.2 (https://www.geneious.com, accessed on 13 May 2024). The thirteen samples of Salsoleae s.l. were selected as ingroups, while two species, *Suaeda glauca* and *Atriplex prostrata*, were used as outgroups. A maximum likelihood (ML) phylogenetic tree was reconstructed using IQ-TREE 2.2.2.6 [49], and the best substitution model, TVM+F+I+R5, was based on the Bayesian information criterion (BIC). A Bayesian inference (BI) analysis was conducted using MrBayes 3.2.7 [50] with the parameters set as follows: ngen = 3,000,000, samplefreq = 200, and burninfrac = 0.25. The trees generated were visualized using FigTree (http://tree.bio.ed.ac.uk/software/figtree/, accessed on 15 May 2024).

## 3. Results

### 3.1. The Features of the S. foliosa, S. tragus, P. affinis, and X. richteri Plastid Genomes

**Figure 2:**
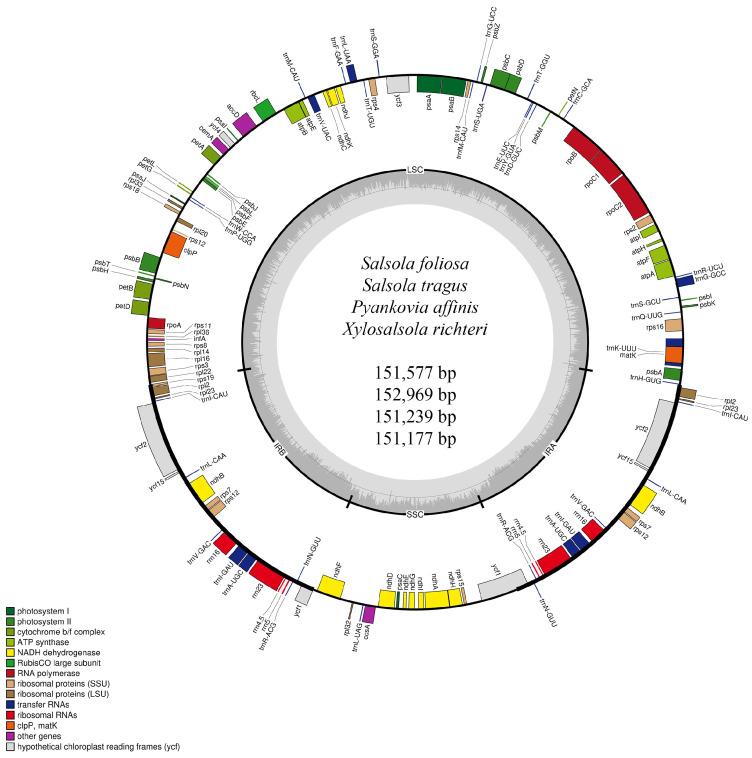
A circular map illustrating the plastid genomes of the species *S. foliosa*, *S. tragus*, *P. affinis*, and *X. richteri* collected in Kazakhstan, with annotated genes color-coded based on their respective functions. Inside the circle, the darker grey shades represent the GC content, while the lighter grey shades represent the AT content. The boundaries of the plastid genome is delineated into LSC, SSC, IRA, and IRB regions.

A total of 24,156,836, 19,894,552, 29,541,702, and 32,239,736 filtered reads were obtained for the plastid genomes of the *S. foliosa*, *S. tragus*, *P. affinis*, and *X. richteri* species, respectively. The final yield of filtered data exceeded 16 GB for all four species. The quality values Q20 were 97.85%, 96.98%, 98.41%, and 97.58% for *S. foliosa*, *S. tragus*, *P. affinis*, and *X. richteri*, respectively. Correspondingly, the Q30 values for the same species were 92.77%, 90.74%, 94.2%, and 92.11%. The total lengths of the assembled *S. foliosa*, *S. tragus*, *P. affinis*, and *X. richteri* plastid genomes were 151,577 bp, 152,969 bp, 151,239 bp, and 151,177 bp, respectively (Figure 2).

The plastid genome sequences of the four species analyzed in this study have been submitted to the GenBank database at the NCBI (National Center for Biotechnology Information) with the following accession numbers: PP754487-PP754490. The examined plastid genome displayed a typical quadripartite structure consisting of paired inverted repeats (46,954–50,400 bp) separated by a large single-copy region (83,324–84,136 bp) and a small single-copy region (18,576–20,087). The inverted repeat regions (42.58–43.17%), large single-copy region (34.10–34.60%), and small single-copy region (28.75–29.76%) exhibited nearly identical GC content values across all four analyzed species. All four examined plastid genomes exhibited a comparable gene content and arrangement. Together, they encompassed 114 unique genes, including 80 protein-coding genes, 30 tRNA genes, and 4 rRNA genes. Moreover, 19 duplicated genes were observed within the inverted region (Table 2).

Among the 114 unique genes identified, 17 contained introns. Specifically, there were eleven protein-coding genes (*rps12*, *rps16*, *rpl16*, *rpoC1*, *atpF*, *ndhA*, *ndhB*, *petB*, *petD*, *clpP*, and *ycf3*), and six tRNA genes (*trnA-UGC*, *trnG-GCC*, *trnI-GAU*, *trnK-UUU*, *trnL-UAA*, and *trnV-UAC*), as detailed in Table 3. The *clpP* and *ycf3* genes each had two introns, while the remaining listed genes featured a single intron. Duplicated copies were detected for eight protein-coding genes (*rps7*, *rps12*, *rpl2*, *rpl23*, *ndhB*, *ycf1*, *ycf2*, and *ycf15*), seven tRNA genes (*trnA-UGC*, *trnI-CAU*, *trnI-GAU*, *trnL-CAA*, *trnN-GUU*, *trnR-ACG*, and *trnV-GAC*), and four rRNA genes (*rrn4.5*, *rrn5*, *rrn16*, and *rrn23*).

### 3.2. Repeat Sequences Analysis

A total of 239, 274, 215, and 259 SSRs were detected in the plastid genome of *S. foliosa*, *S. tragus*, *P. affinis*, and *X. richteri*, respectively. Comparing the frequency of SSRs in the analyzed nucleotide sequences of the plastid genomes revealed that mononucleotides (77.20%) were more prevalent across all the genomes, followed by dinucleotides (16.72%), tetranucleotides (3.34%), and trinucleotides (2.33%). Pentanucleotide repeats (0.20%) were exclusively found in *P. affinis* and *X. richteri*, while hexanucleotide repeats (0.20%) were present only in *P. affinis* and *S. tragus*. The majority of the mononucleotide repeats consisted of A/T, accounting for 97.64% or 744 repeats, with only 2.36% or 18 composed of C/G. Among the dinucleotide repeats, AT/AT sequences constituted 58.79% or 97 repeats, while AG/CT and AC/GT repeats constituted only 36.36% and 4.85%, respectively (Table 4).

**Figure 3:**
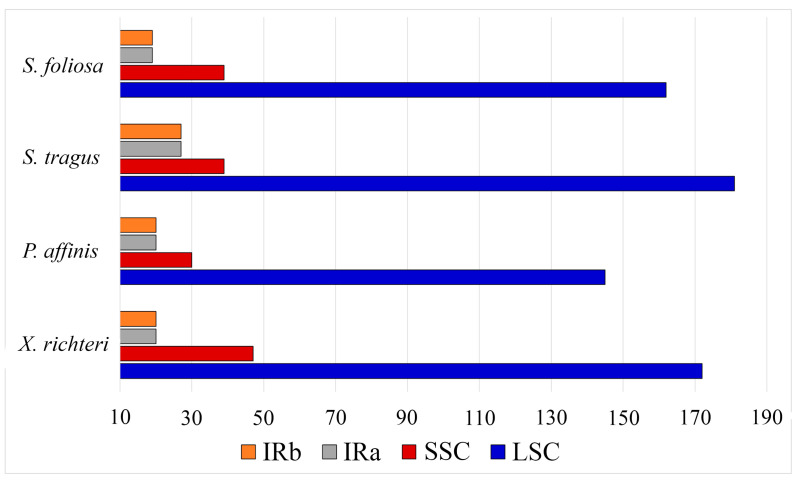
The distribution of identified SSRs across the large single-copy (LSC), small single-copy (SSC), and inverted repeat (IR) regions of the plastid genomes of *S. foliosa*, *S. tragus*, *P. affinis*, and *X. richteri*.

Out of the total 987 SSRs identified, the majority were situated in the intergenic spacer regions (60.98%), followed by the protein-coding gene regions (33.33%) (Supplementary File S1). Additionally, most of the detected SSRs were found in the LSC regions rather than in the SSC or IR regions across the four plastid genomes (Figure 3).

**Figure 4:**
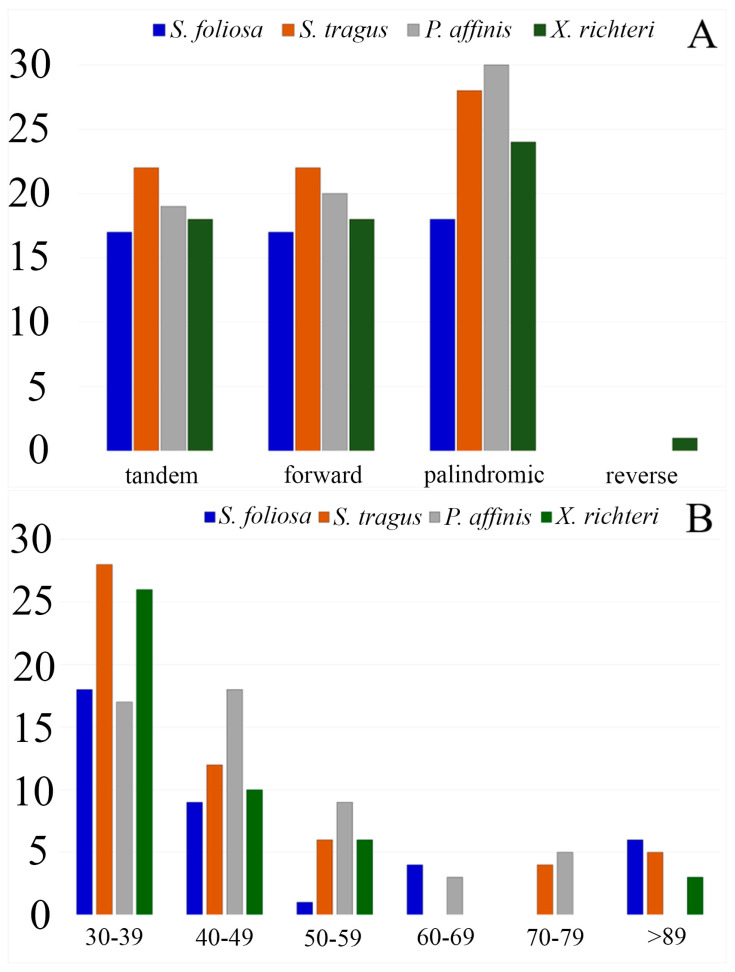
Repeated sequences within the plastid genomes of *S. foliosa*, *S. tragus*, *P. affinis*, and *X. richteri*. (**A**) The overall count of tandem, forward, reverse, and palindromic repeat types. (**B**) The distribution of long repeats was categorized by their respective lengths.

Moreover, the sequences of the four plastid genomes revealed the presence of tandem, forward, reverse, and palindromic repeats. Among these, palindromic repeats were found to be the most prevalent type across the analyzed plastid genomes, while reverse repeats were the least common. In total, 254 repeats were identified, comprising 92 tandem repeats, 88 forward repeats, 100 palindromic repeats, and only 1 reverse repeat (Figure 4A). Repeats ranging from 30 to 39 bp were the most prevalent in all four plastid genomes, with counts of 18, 28, 17, and 26 for *S. foliosa*, *S. tragus*, *P. affinis*, and *X. richteri*, respectively. Repeats with a length of 89 base pairs or more were exclusively detected in the plastid genomes of *S. foliosa*, *S. tragus*, and *X. richteri* (Figure 4B).

### 3.3. Sliding Window Analysis

**Figure 5:**
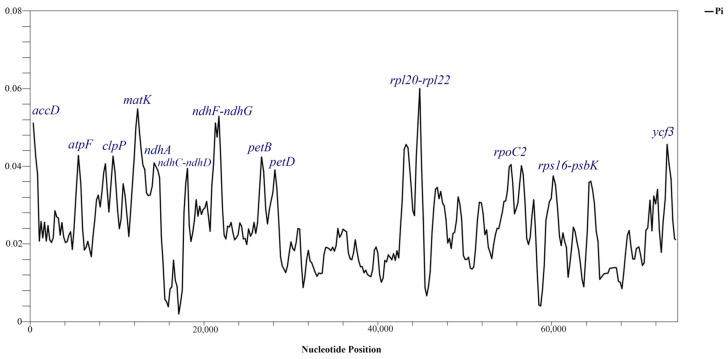
Nucleotide diversity (Pi) values among the protein-coding genes of the 13 species of Salsoleae s.l. based on the sliding window analysis (window length = 600 bp and step size = 200 bp). The vertical axis represents the nucleotide diversity for each window, and the horizontal axis indicates the midpoint position.

To identify the regions with a high variability among the thirteen samples of Salsoleae s.l., we compared the Pi values within coding regions using DnaSP 6. The average nucleotide diversity (Pi) across these species was calculated as 0.02426. We observed nucleotide diversity values ranging from 0 to 0.06000 within 600 base pairs through a sliding window analysis. Thirteen regions exhibited significant variability, characterized by Pi values exceeding 0.03. These regions included *ndhC-ndhD, rps16-psbK, petD, rpoC2, ndhA, petB, clpP, atpF, ycf3, accD, ndhF-ndhG, matK,* and *rpl20-rpl22* (Figure 5). The *rpl20-rpl22* region displayed the highest nucleotide variation (0.06000), as shown in Table 5.

### 3.4. Substitution Rate of Protein-Coding Genes of Salsoleae s.l. Species

**Figure 6:**
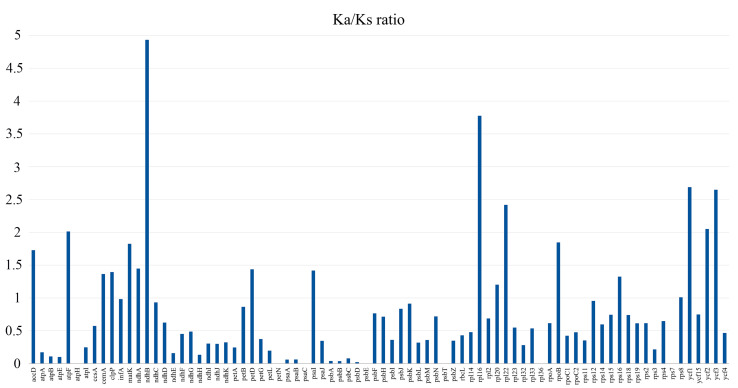
The Ka/Ks ratios of 80 protein-coding genes from 13 samples of Salsoleae s.l. plastid genomes.

Synonymous (Ks) and nonsynonymous (Ka) substitutions, along with their ratio (Ka/Ks), were calculated across the 80 protein-coding genes presented in the plastid genomes of the 13 samples of Salsoleae s.l. (Supplementary File S2). The average Ka/Ks ratio for the 80 protein-coding genes was 0.76. The analysis revealed a zero Ka/Ks ratio for seven genes. However, 55 genes exhibited a Ka/Ks ratio of less than 1, indicating purifying selection. The Ka/Ks ratios of 18 genes exceeded 1, suggesting positive selection (Figure 6).

### 3.5. Phylogenetic Analysis

**Figure 7:**
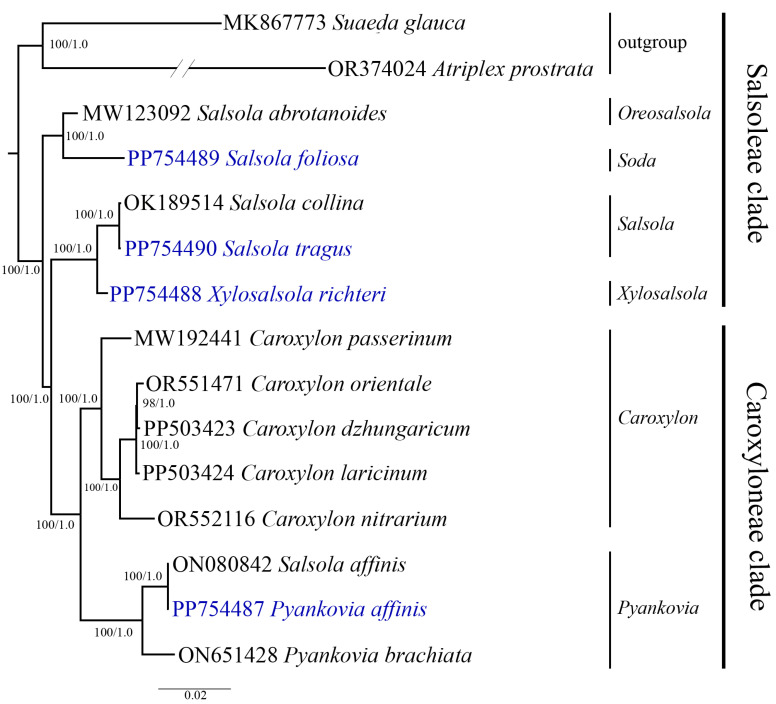
Phylogenetic tree inferred from the nucleotide sequences of the complete plastid genome of 13 samples of Salsoleae s.l. and outgroups, using maximum likelihood (ML) and Bayesian inference (BI) methods. The species collected in this study are highlighted in blue. The numbers in the branch nodes represent ML bootstrap values/BI posterior probabilities values.

The phylogenetic relationships among Salsoleae s.l. species were analyzed based on the nucleotide sequences of complete plastid genome sequences. A multiple sequence alignment was performed on the nucleotide sequences from thirteen Salsoleae s.l. samples and two outgroup species. Maximum likelihood (ML) and Bayesian inference (BI) methods were employed to construct the phylogenetic trees. The resulting trees based on the nucleotide sequences of the complete plastid genome showed a similar topology and clustered the Salsoleae s.l. species into several clusters, which was supported by strong bootstrap and posterior probability values. The analyzed species were divided into two main clusters, corresponding to the Salsoleae and Caroxyloneae clades, and four subclusters. The Salsoleae cluster comprised five species from the genera *Oreosalsola* Akhani, *Soda* (Dumort.) Fourr., *Salsola*, and *Xylosalsola*, while the Caroxyloneae cluster included seven species (or eight samples) from the genera *Caroxylon* Thunb. and *Pyankovia*. The species *S. foliosa* was clustered with *S. abrotanoides* in a separate subcluster. *S. tragus* and *X. richteri*, along with the GenBank sample *S. collina*, formed a second subcluster in the Salsoleae clade, indicating a close relationship among these species. The species from *Caroxylon* grouped together and made a third subcluster. *P. affinis*, the next species analyzed in this study, grouped with *S. affinis* and *P. bachiata* obtained from GenBank in the Caroxyloneae clade. Each clade was represented by two subclusters (Figure 7).

## 4. Discussion

In this study, we sequenced, assembled, and annotated the plastid genomes of *Salsola foliosa*, *Salsola tragus*, *Pyankovia affinis*, and *Xylosalsola richteri* species from Kazakhstan. These species’ complete plastid genome sizes ranged from 151,177 bp to 152,969 bp, which was consistent with the genome sizes of other Amaranthaceae species [51,52,53]. The total GC content varied slightly, ranging from 36.26% in *P. affinis* to 36.60% in *S. tragus* and *X. richteri*. A similar GC content has been observed in other angiosperm plastid genomes [54,55]. The plastid genomes of *S. foliosa*, *S. tragus*, *P. affinis*, and *X. richteri* are highly conserved, comprising four parts and featuring an identical gene content and gene order. The gene loss and genome rearrangement documented in various angiosperm species [56,57] were not observed in the plastomes of *S. foliosa*, *S. tragus*, *P. affinis*, and *X. richteri*. A total of 114 unique genes were annotated, including 80 protein-coding genes, 30 tRNA genes, and 4 rRNA genes (Table 3).

The thirteen regions displaying relatively high variability, specifically *ndhC-ndhD*, *rps16-psbK*, *petD*, *rpoC2*, *ndhA*, *petB*, *clpP*, *atpF*, *ycf3*, *accD*, *ndhF-ndhG*, *matK*, and *rpl20-rpl22*, were identified by Pi values exceeding 0.03 (Table 5). These regions exhibited a substantially higher nucleotide diversity when compared to *psbB–psbH* and *rbcL*, which were previously employed in phylogenetic analyses for the revised classification of Salsoleae s.l. [10,11]. In this study, the *matK* gene, recognized as a core barcode by the CBOL [58], was identified as a region demonstrating a relatively high variability, with a Pi value of 0.05478. The thirteen regions identified to exhibit a relatively high variability could serve as potential DNA barcoding markers and valuable resources in assessing the taxonomy of Salsoleae s.l.

The Ka/Ks ratio provides a valuable measure of selection pressure on individual protein-coding genes. A Ka/Ks ratio below 1 signifies negative or purifying selection, while a ratio of 1 indicates neutral selection, and a Ka/Ks ratio exceeding 1 indicates positive selection [59]. In this study, the Ka/Ks analysis suggested that the majority (55) of the genes were under negative selection, while 18 were identified as being under positive selection (Figure 6). These findings are consistent with reports indicating that the prevalence of protein-coding genes undergoing negative selection is common in angiosperms [55,60,61]. Notably, the *ndhB* gene exhibited the highest Ka/Ks ratio (4.9), suggesting significant positive selection in the analyzed Salsoleae s.l. species, a conclusion supported by earlier studies [60,61].

SSRs extracted from plastid genomes are variable molecular markers known for their high levels of polymorphism and stability, as documented in the literature [62,63]. Plastid genome SSRs are widely utilized in population genetics and evolutionary studies due to their effectiveness in revealing levels of genetic diversity and population structures [64,65]. In this study, a total of 987 SSRs were identified across the four analyzed plastid genomes (Table 4). Mononucleotide repeats were the most abundant, constituting 77.20% of the total SSRs, followed by dinucleotide, tetranucleotide, trinucleotide, pentanucleotide, and hexanucleotide repeats. Previous studies have reported similar results [66,67]. Most mononucleotide and dinucleotide repeats consisted of A/T (97.64%) and AT/AT (58.79%). The obtained results aligned with prior research indicating that plastid genome SSRs are primarily composed of polyadenine (poly A) or polythymine (poly T) repeats, with infrequent occurrences of tandem guanine (G) or cytosine (C) repeats [68,69]. The higher abundance of tetranucleotide repeats compared to trinucleotide, pentanucleotide, and hexanucleotide repeats is consistent with findings in other species [70]. Pentanucleotide and hexanucleotide repeats were rare across the plastid genomes of *S. foliosa*, *S. tragus*, *P. affinis*, and *X. richteri*, mirroring the results observed in *Caroxylon* species from Amaranthaceae [71]. These newly acquired plastid genome SSRs have the potential to bridge the gap caused by the lack of species-specific SSRs designed for the *Salsola*, *Xylosalsola*, and *Pyankovia* taxa.

Complete plastid genome nucleotide sequences offer invaluable data for elucidating the phylogenetic relationships among plants and are widely utilized for this purpose [72,73]. Plastid genome nucleotide sequences have been utilized to assess the phylogenetic relationships among *Caroxylon* species and other members of the tribe Salsoleae s.l., for which plastid genome data are accessible in GenBank [71]. Previously, extensive phylogenetic studies of Salsoleae s.l. have been conducted, employing both nuclear genes and several plastid genome genes to gain a comprehensive understanding of the evolutionary relationships within this tribe [10,11]. Despite these efforts, the taxonomy of genera such as *Salsola*, *Pyankovia*, and *Xylosalsola* still presents challenges and remains unclear. This study employed nucleotide sequences derived from complete plastid genomes to elucidate the phylogenetic relationships among the analyzed species. In total, thirteen Salsoleae s.l. plastid genomes, including four genomes reported in this study, were analyzed in addition to two outgroups (Figure 7). The dendrogram based on full plastid genomes formed two clear clusters and four subclusters. According to Akhani et al. [10], the first two clusters represented the Salsoleae tribe clade, and the other two represented the Caroxyloneae tribe clade. Within the Caroxyloneae tribe clade, the dendrogram clearly grouped five species from the genus *Caroxylon* (Figure 7). Previously, this group of species from this genus was assessed using full plastid sequences [71], confirming their taxonomic position within the Caroxyloneae clade. The other cluster of the clade consisted of three species of *Pyankovia*, including *P. affinis*. The distinct separation of *P. affinis* from three other species described in this study was congruent with a morphological description of these taxa (Figure 1). The morphology of *P. affinis* is drastically different from that of the other three species, as it has fleshy leaves with obtuse and semi-rolled forms (Figure 1G).

In the Salsoleae tribe clade, the species *S. abrotanoides*, which was accepted as *Oreosalsola abrotanoides* (Bunge) Akhani [74], and *S. foliosa*, which was recognized as *Soda foliosa* (L.) Akhani by Rudov et al. [75], were clearly distinguished from the subcluster consisting of *X. richteri*, *S. tragus*, and *S. collina* (Figure 7). The taxonomic position of *S. foliosa* within this clade and the separation from *S. tragus* and *X. richteri* also reflects a number of distinct morphological features of this species, such as bare and branched stems and leaves that are club-shaped and arched upwards (Figure 1E). The clustering of the three latter species of the clade agrees well with Wen and co-authors [11], as the topology of the two dendrograms for these species is similar. Still, the results provided by Akhani and co-authors [10] suggested that *X. richteri* belongs to the genus *Xylosalsola*. Therefore, the generated ML/BI dendrogram indicated that *S. collina* and *S. tragus* belong to the genus *Salsola* in the Kali clade and confirmed that *X. richteri* is distantly separated from these two species as a representative of the genus *Xylosalsola*. The separation of *S. tragus* and *X. richteri* is also easily visible morphologically. The bracts of *S. tragus* are pointed with a spine at the apex (Figure 1F), unlike in *X. richteri*, where the short-pointed leaves are filamentous or linear (Figure 1H). While our results provide some insights, additional plastid genome data from *Salsola* and *Xylosalsola* species are necessary to clarify the relationships between these genera thoroughly.

In general, the generated complete plastid sequences for these four species of Salsoleae s.l. may have important biological significance in further taxonomic, evolutionary, and conservation studies in these taxa. For instance, here, we identified more than 200 candidate SSR markers for each of the studied species, which can potentially be efficiently used for the extraction of information on most polymorphic SSRs as informative tools in genetic studies of these species, in the evaluation of the genetic diversity within and between populations, and in the identification of most diverse populations within a species for the development of robust strategies in germplasm conservation projects. Although our current study examined only a limited number of species, ongoing advancements in sequencing technologies are poised to significantly expand the pool of sequenced plastid genomes. Consequently, the data obtained in this study hold promise as a valuable resource for future phylogenetic analyses within the tribe of Salsoleae s.l.

## 5. Conclusions

The assembled plastid genomes of *S. foliosa*, *S. tragus*, *P. affinis*, and *X. richteri* had lengths of 151,577 bp, 152,969 bp, 151,239 bp, and 151,177 bp, respectively. Thirteen regions, including *ndhC-ndhD*, *rps16-psbK*, *petD*, *rpoC2*, *ndhA*, *petB*, *clpP*, *atpF*, *ycf3*, *accD*, *ndhF-ndhG*, *matK*, and *rpl20-rpl22*, exhibited a relatively high nucleotide diversity. These genes could serve as potential DNA-barcoding markers for Salsoleae tribe species. Among 55 genes, the Ka/Ks ratio was <1, indicating purifying selection, while for 18 genes, the ratio was >1, suggesting positive selection; the *ndhB* gene had the highest Ka/Ks ratio (4.9). The plastid genomes of *S. foliosa*, *S. tragus*, *P. affinis*, and *X. richteri* contained 239, 274, 215, and 259 SSRs, respectively, with the majority located in intergenic spacer regions (60.98%). The maximum likelihood and Bayesian inference phylogenetic tree separated thirteen plastid genomes into four distinct clusters corresponding to six genera in the Salsoleae and Caroxyloneae clades. It was suggested that the generated sequences of the four complete plastid genomes comprise a valuable resource for future phylogenetic analyses within Salsoleae s.l.

## Figures and Tables

**Table 1:** The information on the collected places of *S. foliosa*, *S. tragus*, *P. affinis*, and *X. richteri* plant materials.

Species	*S. foliosa*	*S. tragus*	*P. affinis*	*X. richteri*
Collected place	West Kazakhstan region, Borly district	Kyzylorda region, Zhalagash district	Zhetysu region, Panfilov district	Kyzylorda region, Zhanakorgan district
GPS coordinates	51.29	45.08	44.17	44.33
	53.36	64.78	79.53	66.21
	82 m a.s.l.	110 m a.s.l.	890 m a.s.l.	150 m a.s.l.

**Table 2:** Plastid genome features of *S. foliosa*, *S. tragus*, *P. affinis*, and *X. richteri*.

	*S. foliosa*	*S. tragus*	*P. affinis*	*X. richteri*
GenBank numbers	PP754489	PP754490	PP754487	PP754488
Genome size (bp)	151,577	152,969	151,239	151,177
LSC (bp)	83,871	83,993	83,324	84,136
SSC (bp)	18,962	18,576	18,739	20,087
IR (bp)	48,744	50,400	49,176	46,954
Number of total genes	133	133	133	133
Protein-coding genes	80	80	80	80
tRNAs	30	30	30	30
rRNAs	4	4	4	4
Total GC content (%)	36.55	36.60	36.26	36.60
LSC GC content (%)	34.45	34.60	34.10	34.57
SSC GC content (%)	29.59	29.38	28.75	29.76
IR GC content (%)	42.88	42.58	42.77	43.17

**Table 3:** Annotated genes within the *S. foliosa*, *S. tragus*, *P. affinis*, and *X. richteri* plastid genomes.

Category	Group of Genes	Name of Genes
Self-replication	Ribosomal RNA	*rrn4.5* (2), *rrn5* (2), *rrn16* (2), *rrn23* (2)
	Transfer RNA	*trnA-UGC* * (2), *trnC-GCA*, *trnD-GUC*, *trnE-UUC*, *trnF-GAA*, *trnfM-CAU*, *trnG-GCC* *, *trnG-UCC*, *trnH-GUG*, *trnI-CAU* (2), *trnI-GAU* * (2), *trnK-UUU* *, *trnL-CAA* (2), *trnL-UAA* *, *trnL-UAG*, *trnM-CAU*, *trnN-GUU* (2), *trnP-UGG*, *trnQ-UUG*, *trnR-ACG* (2), *trnR-UCU*, *trnS-GCU*, *trnS-GGA*, *trnS-UGA*, *trnT-GGU*, *trnT-UGU*, *trnV-GAC* (2), *trnV-UAC* *, *trnW-CCA*, *trnY-GUA*
	Small subunit of ribosome	*rps2*, *rps3*, *rps4*, *rps7* (2), *rps8*, *rps11*, *rps12* * (2), *rps14*, *rps15*, *rps16* *, *rps18*, *rps19*
	Large subunit of ribosome	*rpl2* (2), *rpl14*, *rpl16* *, *rpl20*, *rpl22*, *rpl23* (2), *rpl32*, *rpl33*, *rpl36*
	RNA polymerase	*rpoA*, *rpoB*, *rpoC1* *, *rpoC2*
	Translation initiation factor	*infA*
Photosynthesis	ATP synthase	*atpA*, *atpB*, *atpE*, *atpF* *, *atpH*, *atpI*
	NADH dehydrogenase	*ndhA* *, *ndhB* * (2), *ndhC*, *ndhD*, *ndhE*, *ndhF*, *ndhG*, *ndhH*, *ndhI*, *ndhJ*, *ndhK*
	Subunits of cytochrome	*petA*, *petB* *, *petD* *, *petG*, *petL*, *petN*
	Photosystem I	*psaA*, *psaB*, *psaC*, *psaI*, *psaJ*
	Photosystem II	*psbA*, *psbB*, *psbC*, *psbD*, *psbE*, *psbF*, *psbH*, *psbI*, *psbJ*, *psbK*, *psbL*, *psbM*, *psbN*, *psbT*, *psbZ*
	Rubisco	*rbcL*
Other genes	Maturase	*matK*
	Protease	*clpP* **
	Envelope membrane protein	*cemA*
	Subunit of acetyl-CoA-carboxylase	*accD*
	C-type cytochrome synthesis gene	*ccsA*
Genes of unknown function	Hypothetical chloroplast reading frames	*ycf1* (2), *ycf2* (2), *ycf3* **, *ycf4*, *ycf15* (2)

* One-intron-containing genes; ** two-intron-containing genes; (2) duplicated genes.

**Table 4:** SSRs detected in the plastid genomes of *S. foliosa*, *S. tragus*, *P. affinis*, and *X. richteri*.

Type	Repeats	*S. foliosa*	*S. tragus*	*P. affinis*	*X. richteri*	Total	%
Mono	A/T	180	207	160	197	744	77.20
	C/G	3	6	5	4	18	
Di	AC/GT	2	1	4	1	8	16.72
	AG/CT	16	14	14	16	60	
	AT/AT	28	24	18	27	97	
Tri	AAG/CTT	1	1	2	1	5	2.33
	AAT/ATT	1	12	2	3	18	
Tetra	AAAC/GTTT	1	1	0	1	3	3.34
	AAAG/CTTT	1	1	2	1	5	
	AAAT/ATTT	1	0	1	1	3	
	AAGG/CCTT	0	1	0	1	2	
	AATG/ATTC	0	1	0	1	2	
	AATT/AATT	3	2	3	2	10	
	ACCT/AGGT	2	2	2	2	8	
Penta	AAATG/ATTTC	0	0	1	1	2	0.20
Hexa	AAAATT/AATTTT	0	1	0	0	1	0.20
	AATCCG/ATTCGG	0	0	1	0	1	
Total		239	274	215	259	987	100

**Table 5:** Highly variable regions in the protein-coding genes of the 13 Salsoleae s.l. species.

Variable Region	Length	Variable Sites	Parsimony-Informative Sites	Nucleotide Diversity
*ndhC-ndhD*	600	75	44	0.03947
*rps16-psbK*	609	82	47	0.03778
*petD*	688	90	44	0.03905
*rpoC2*	606	86	45	0.04046
*ndhA*	864	83	45	0.04086
*petB*	744	98	43	0.04238
*clpP*	723	96	46	0.04262
*atpF*	779	87	48	0.04278
*ycf3*	669	96	50	0.04564
*accD*	729	110	53	0.05114
*ndhF-ndhG*	606	106	63	0.05288
*matK*	606	106	65	0.05478
*rpl20-rpl22*	606	133	58	0.06000

## Data Availability

The plastid genome sequences of the four species analyzed in this study have been submitted to the GenBank database at the NCBI (National Center for Biotechnology Information) with the following accession numbers: PP754487-PP754490.

## Supplementary Materials

The following supporting information can be downloaded at: https://www.mdpi.com/article/10.3390/biom14080890/s1, Supplementary File S1: List of simple sequence repeats identified in the plastid genomes of *S. foliosa*, *S. tragus*, *P. affinis*, and *X. richteri*. Supplementary File S2: The Ka/Ks analysis of 80 protein-coding genes from Salsoleae s.l. plastid genomes.
